# Catheter ablation for patients with atrial fibrillation and heart failure with reduced and preserved ejection fraction: insights from the KiCS-AF multicentre cohort study

**DOI:** 10.1093/europace/euac108

**Published:** 2022-07-19

**Authors:** Yasuyuki Shiraishi, Shun Kohsaka, Nobuhiro Ikemura, Takehiro Kimura, Yoshinori Katsumata, Kojiro Tanimoto, Masahiro Suzuki, Ikuko Ueda, Keiichi Fukuda, Seiji Takatsuki

**Affiliations:** Department of Cardiology, Keio University School of Medicine, Tokyo, Japan; Department of Cardiology, Keio University School of Medicine, Tokyo, Japan; Department of Cardiology, Keio University School of Medicine, Tokyo, Japan; Department of Cardiology, Keio University School of Medicine, Tokyo, Japan; Department of Cardiology, Keio University School of Medicine, Tokyo, Japan; Department of Cardiology, National Hospital Organization Tokyo Medical Center, Tokyo, Japan; Department of Cardiology, National Hospital Organization Saitama Hospital, Saitama, Japan; Department of Cardiology, Keio University School of Medicine, Tokyo, Japan; Department of Cardiology, Keio University School of Medicine, Tokyo, Japan; Department of Cardiology, Keio University School of Medicine, Tokyo, Japan

**Keywords:** Atrial fibrillation, Heart failure, Catheter ablation, Quality of life, Heart failure hospitalization

## Abstract

**Aims:**

The usefulness of catheter ablation (CA) for atrial fibrillation (AF) across a broad spectrum of heart failure (HF) patients remains to be established. We assessed the association of CA with both health-related quality of life (QoL) and cardiovascular events among HF patients with reduced and preserved left ventricular ejection fraction (LVEF) in an ‘all-comer’ outpatient-based AF registry.

**Methods and results:**

Of 3303 patients with AF consecutively enrolled in a retrospective multicentre registry that mandated the Atrial Fibrillation Effect on QualiTy-of-life (AFEQT) questionnaire at registration and 1-year follow-up, we extracted data from 530 patients complicating clinical HF. The association between CA and both 1-year change in AFEQT Overall Summary (AFEQT-OS) scores and 2-year composite clinical outcomes (including all-cause death, stroke, and HF hospitalization) was assessed by multivariable analyses. The median duration of AF was 108 days (52–218 days), and 83.4% had LVEF >35%. Overall, 75 patients (14.2%) underwent CA for AF within 1-year after registration. At 1-year follow-up, 67.2% in the ablation group showed clinically meaningful improvements of ≥ 5 points in AFEQT-OS score than 47.8% in the non-ablation group {adjusted odds ratio, 2.03 [95% confidence interval (CI): 1.13–3.64], *P* = 0.017}. Furthermore, the composite endpoint of all-cause death, stroke, and HF hospitalization occurred less frequently in the ablation group than the non-ablation group [adjusted hazard ratio, 0.27 (95% CI: 0.09–0.86), *P* = 0.027].

**Conclusion:**

Among AF-HF patients, CA was associated with improved QoL and lower risk of cardiovascular events against drug therapy alone, even for patients with mildly reduced and preserved LVEF.

**What’s new?**

- Satisfactory restoration and maintenance of sinus rhythm driven by catheter ablation (CA) in highly selected patients with atrial fibrillation (AF) and heart failure (HF), especially with reduced left ventricular ejection fraction (LVEF), is known to improve patient outcomes when compared to drug therapy alone.
- There is limited evidence supporting the superiority of CA over drug therapy in terms of patient outcomes across a broad range of patients with concomitant AF and HF, especially those with mildly reduced and preserved LVEF.
- In 530 patients with AF and HF (83.4% with LVEF >35%), CA therapy was associated with improved quality of life and lower risk of cardiovascular events when referenced to drug therapy alone, irrespective of LVEF.
- This finding from a registry-based cohort study suggests both symptomatic and prognostic benefits of CA for AF, even in HF patients with mildly reduced or preserved LVEF.

## Introduction

Heart failure (HF) and atrial fibrillation (AF) often coexist,^1^ i.e. 30–40% of HF cases are complicated by AF,^2,3^ and AF increases the risk of thromboembolism, hospitalization for HF, and death.^4,5^ When developing an effective treatment strategy for AF, satisfactory restoration and maintenance of sinus rhythm, mainly driven by catheter ablation (CA) in AF patients with HF, especially with reduced ejection fraction (HFrEF), is known to improve patient outcomes when compared to drug therapy alone.^6,7^

In the absence of sufficient evidence on the prognostic value of AF ablation in patients with HF with preserved ejection fraction (HFpEF), the CA vs. Antiarrhythmic Drug Therapy for AF (CABANA) trial implied a symptomatic and prognostic benefit from CA.^8^ More recently, the Early Treatment of Atrial Fibrillation for Stroke Prevention (EAST-AFNET4) trial showed that early rhythm control strategy, inclusive of CA, reduced cardiovascular events in patients with AF diagnosis within 12 months, and HF in which left ventricular ejection fraction (LVEF) was largely preserved.^9^ However, there is limited evidence supporting the superiority of CA over drug therapy in terms of patient outcomes across a broader range of patients with concomitant AF and HF, especially those with mildly reduced and preserved LVEF.^10,11^ Recently, the prevalence of HFpEF has increased due to changes in population demographics and the prevalence and treatment of risk factors for HF. Causal links between AF and HF may differ between HFrEF and HFpEF, which is possibly a clinical sign of advanced stage HF with a relatively homogeneous elevation of biomarkers, while AF and HFpEF overlap in the underlying pathology and often progress parallelly.^12,13^

Herein, we aimed to investigate the association between CA and drug therapy alone with patient outcomes, including health-related quality of life (QoL) and cardiovascular events, in AF patients complicated with HF (e.g. HFrEF and HFpEF), using a multicentre cohort registry that mainly included patients with AF in its early stage.

## Methods

### Study cohort

The rationale and design of the Keio Interhospital Cardiovascular Studies–Atrial Fibrillation (KiCS-AF) registry have been described previously.^14^ Briefly, the KiCS-AF is a multicenter, registry-based retrospective cohort study designed to collect clinical variables and outcome data from consecutive patients with AF who were newly diagnosed or referred to an outpatient clinic at each of the 11 participating tertiary care hospitals within the Kanto area of Japan. To investigate the association between treatment intervention and health-related QoL, the registry included patients with AF newly referred to the network hospitals within the previous 6 months. Approximately 150 variables linked to the patients’ background, symptoms, prior and current drug use, electrocardiography (ECG) and echocardiography results, and blood sampling test results were collected for each patient. Yearly follow-up examinations were conducted for all patients by mail, phone interviews, and chart reviews. Dedicated study coordinators updated the status of major cardiovascular events and performed the procedures. Data quality assurance was achieved through systematic validation that highlighted outliers and data completeness. The clinical research coordinators in each institution answered all inquiries regarding data entry. To ensure consecutive case enrolment, the senior study coordinator (I.U.) and investigator (S.K.) performed on-site auditing to ensure proper registration of each eligible patient. The protocol was approved by the ethical review board of each institution, and all participants provided written informed consent. Almost all of the approached patients agreed to participate in the present study.

### Assessment of health-related QoL

In addition to traditional data collected by healthcare providers, the KiCS-AF also collected patient-reported outcomes using the internationally validated Atrial Fibrillation Effect on QualiTy-of-life (AFEQT; http://www.afeqt.org).^15^ Patients were requested to complete the AFEQT questionnaire at registration and follow-up visits (e.g. 1 year after registration) or by mail. The AFEQT is a 20-item questionnaire that maps four domains of AF-related QoL, including symptoms, daily activities, treatment concerns, and treatment satisfaction, using a 7-point Likert response scale. An overall summary score can be calculated from the first three domains and ranges from 0 to 100 [100, best possible health status (no impairment); 0, worst health status]. A recent analysis has suggested that a 5-point change in the AFEQT Overall Summary (AFEQT-OS) score is observed among patients who change by one class of European Heart Rhythm Association functional status and is a clinically meaningful difference.^16^ We also defined the grade of changes in the AFEQT-OS score as follows: large improvement (≥15 points), moderate improvement (10–15 points), small improvement (5–10 points), no change (−5 to 5 points), small worsening (−10 to −5 points), moderate worsening (−15 to −10 points), and large worsening (≤ −15 points). A culturally and linguistically translated version of the AFEQT for Japan was used.

### Analytic cohort

Of the 3303 patients with AF registered from 2012 to 2017, 535 (16.2%) patients had clinical HF at baseline. After excluding five patients whose follow-up information was unavailable, 530 patients were analyzed in the present study. In the analysis of health-related QoL and cardiovascular events, patients were divided into two groups according to whether they had CA within 1 year or within 2 years, respectively.

### Definitions of variables and outcomes

The history of HF was coded based on documentation of medical records. Clinical HF was defined based on the Framingham criteria (major and minor symptoms or signs of HF) and/or possible clinical improvement on HF treatment. Atrial fibrillation type was classified as first diagnosed, paroxysmal, persistent, or permanent according to the 2010 ESC guidelines for the management of AF.^17^ In this study, first diagnosed and permanent AF were merged into ‘others’ as the number of first diagnosed AF was very small (*n* = 18). The echocardiographic variables were obtained from the evaluation of left ventricular function, including the quantification of LVEF via Simpson’s method in the 4-chamber and 2-chamber views and the left atrial diameter measured in the parasternal long-axis view. To obtain echocardiographic parameters, at least five consecutive heartbeats were recorded and averaged for each parameter.

As for health-related QoL, the improvement with five or more points in the AFEQT-OS score between baseline and 1-year visit was assessed in the present study. In addition, the studied clinical outcome was a composite of all-cause death, stroke, or HF hospitalization. Another composite outcome of all-cause death or HF hospitalization according to the Catheter Ablation for Atrial Fibrillation with Heart Failure (CASTLE-AF) trial was also assessed.^6^

### Statistical analysis

With respect to descriptive statistics, continuous variables are presented as the median and interquartile range (IQR), whereas categorical variables are presented as frequency and percentage. For baseline characteristics, the ablation and non-ablation groups were compared using the Mann–Whitney *U* test for continuous variables and Pearson’s χ^2^ test for categorical variables. Associations between covariates and improvements in health-related QoL (changes in AFEQT-OS score ≥5 points) and composite clinical events were evaluated using both univariate and multivariable models. Unadjusted and adjusted odds ratios (ORs) or hazard ratios (HRs) and their 95% confidence intervals (CIs) were estimated.

First, models were fit via complete case analysis, wherein the patients with missing data on at least one of the variables in multiple logistic regression were excluded from the analyses. Next, all patients were included in the multivariate regression using multiple imputation, which is based on Bayesian theory and is a principled method under missing at random (MAR) as a missing mechanism. Missing at random assumes that missing values can be explained using all observed data. Multiple imputation by chained equations (MICE) algorithm was applied, in which linear regression, logistic, and discriminant imputation methods were used for continuous, binary, and multinomial variables, respectively. The number of burn-in iterations between imputations was 10, and the number of imputations was 10 in MICE. Rubin’s rule was used to combine estimates and their precision from the analysis of multiply imputed data.

We constructed logistic regression models with generalized estimating equations to account for the clustering of patients within sites. Whether the AFEQT-OS score improved to ≥5 points was entered as a dependent variable. The model was adjusted for clinically relevant factors: age, sex, AF type (paroxysmal vs. others), coronary artery disease, AFEQT-OS score at baseline, and CA. We then performed a subgroup analysis for LVEF (<50% vs. ≥50%). In addition, a sensitivity analysis was performed to exclude patients without a prescription of diuretics at baseline, as these patients might have difficulty in correctly judging their signs and symptoms derived from HF but not AF.

Survival curves were calculated using Kaplan–Meier estimates and were analyzed using the log-rank test. Multivariable Cox proportional hazard models were used to identify independent predictors of each endpoint. Models were adjusted for CHA_2_DS_2_-VASc score, AF type (paroxysmal vs. others), LVEF, eGFR (<60 vs. ≥60 mL/min/1.73 m^2^), anaemia (haemoglobin level <13 g/dL for men and <12 g/dL for women according to the WHO scientific definition), and CA. We then performed a sensitivity analysis to exclude patients who were not prescribed diuretics at baseline.

All probability values were two-tailed, and values of *P* < 0.05 were considered statistically significant. All statistical analyses were performed using IBM SPSS Statistics for Windows, version 27 (IBM Corp., Armonk, NY, USA).

## Results

### Baseline characteristics

In the entire cohort, the median duration of AF was 108 days (52–218 days) for the studied patients, and 83.4% had LVEF >35%, which was the exclusion criterion for the CASTLE-AF trial. *Table 1* shows the patient characteristics in the ablation and non-ablation groups. Compared with 455 (85.8%) patients in the non-ablation group, 75 (14.2%) patients in the ablation group were likely to be younger [median (IQR) age, 67 (59–73) years vs. 76 (67–81) years; *P* <0.001] and men (77% vs. 59%; *P* = 0.002), and had a lower CHA_2_DS_2_-VASc score and higher haemoglobin level (both *P* <0.001). Notably, there was no difference in LVEF between the two groups. As for the standard medical therapy for HF, diuretics were more frequently prescribed in the non-ablation group than in the ablation group.

**Table 1:** Baseline characteristics in the ablation group vs. the non-ablation group

	Missing data, *n* (%)	Ablation *n* = 75	Non-ablation *n* = 455	*P*-value
Age, years	0 (0)	67 (59–73)	76 (67–81)	<0.001
Men, *n* (%)	0 (0)	58 (77)	267 (59)	0.002
Body mass index, kg/m^2^	3 (0.1)	23.1 (21.1–25.8)	22.4 (20.1–25.4)	0.135
Systolic BP, mm Hg	95 (17.9)	124 (111–138)	123 (110–135)	0.414
Heart rate, bpm	22 (4.2)	80 (68–99)	80 (67–92)	0.347
LVEF, %	40 (7.5)	55 (46–60)	55 (41–60)	0.466
LVDD, mm	40 (7.5)	49 (45–53)	49 (44–55)	0.945
LVSD, mm	46 (8.7)	33 (30–41)	34 (29–41)	0.781
LAD, mm	35 (6.6)	42 (38–46)	46 (41–51)	<0.001
AF duration, days	94 (17.7)	186 (58–690)	101 (51–186)	0.007
Type of AF	4 (0.1)			0.026
ȃParoxysmal, *n* (%)		23 (31)	102 (23)	
ȃPersistent, *n* (%)		35 (47)	158 (35)	
ȃOthers, *n* (%)		17 (22)	195 (42)	
Comorbidities				
ȃCoronary artery disease, *n* (%)	0 (0)	9 (12)	69 (15)	0.469
ȃHypertension, *n* (%)	0 (0)	51 (68)	304 (67)	0.84
ȃDiabetes mellitus, *n* (%)	0 (0)	15 (20)	112 (25)	0.386
ȃDyslipidemia, *n* (%)	0 (0)	36 (48)	184 (40)	0.218
ȃStroke, *n* (%)	0 (0)	5 (7)	55 (12)	0.17
ȃPeripheral artery disease, *n* (%)	0 (0)	2 (3)	25 (6)	0.301
ȃCOPD, *n* (%)	0 (0)	2 (3)	20 (4)	0.487
ȃCHADS_2_-VASc score	0 (0)	3 (2–4)	4 (3–5)	<0.001
Laboratory findings				
ȃHaemoglobin, g/dL	14 (2.6)	14.2 (13.1–15.0)	13.1 (11.9–14.5)	<0.001
ȃeGFR, mL/min/1.73 m^2^	11 (2.1)	56.5 (47.0–66.8)	52.9 (43.4–64.4)	0.098
ȃBNP, pg/mL	160 (30.2)	160 (98–338)	209 (106–380)	0.098
Medical therapy				
ȃDiuretic, *n* (%)	0 (0)	48 (64)	363 (80)	0.002
ȃACEI or ARB, *n* (%)	0 (0)	43 (57)	240 (53)	0.472
ȃBeta blocker, *n* (%)	0 (0)	58 (77)	347 (76)	0.864
ȃDigoxin, *n* (%)	0 (0)	10 (13)	74 (16)	0.515
ȃAntiarrhythmics, *n* (%)	0 (0)	12 (16)	36 (8)	0.024
ȃOral anticoagulant, *n* (%)	0 (0)	72 (96)	423 (93)	0.355
AFEQT scores at baseline	1 (0.1)			
ȃOverall summary score		71.3 (61.8–82.4)	76.3 (63.0–87.0)	0.035
ȃSymptom		79.2 (66.7–91.7)	87.5 (63.0–87.0)	0.013
ȃDaily activity		68.8 (52.1–79.2)	68.8 (52.1–85.4)	0.219
ȃTreatment concern		76.7 (66.7–86.1)	83.3 (69.4–91.7)	0.024
ȃTreatment satisfaction		66.7 (50–66.7)	66.7 (66.7–83.3)	0.001

BP, blood pressure; LVEF, left ventricular ejection fraction; LVDD, left ventricular diastolic diameter; LVSD, left ventricular systolic diameter; AF, atrial fibrillation; COPD, chronic obstructive pulmonary disease; eGFR, estimated glomerular filtration ratio; BNP, B-type natriuretic peptide; ACEI, angiotensin-converting enzyme inhibitor; ARB, angiotensin receptor blocker.

The AFEQT-OS score [median (IQR): 71.3 (61.8–82.4) vs. 76.3 (63.0–87.0); *P* = 0.035], as well as each domain of the AFEQT score, except daily activity, were consistently lower in the ablation group than in the non-ablation group.

### Changes in the AFEQT-OS score after 1-year

**Figure 1:**
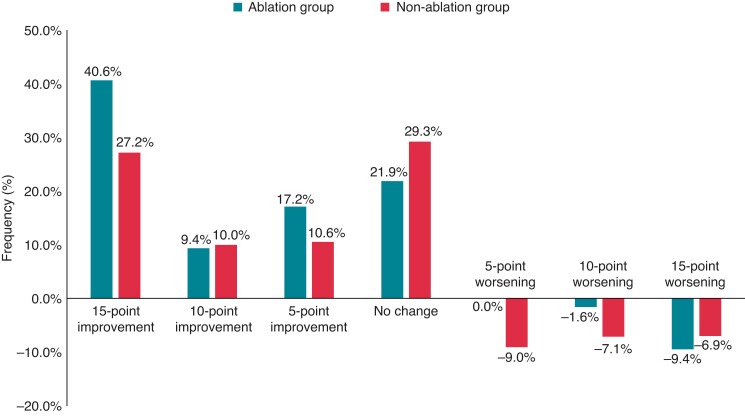
Proportion of the change in the AFEQT-OS score by the application of catheter ablation. Green and red colours show the ablation and non-ablation groups, respectively.

Of the studied patients, 443 (83.6%) underwent QoL assessment using the AFEQT questionnaire at both baseline and 1-year visits. At 1 year after enrolment, 67.2% of patients in the ablation group had an improvement of 5 or more points in the AFEQT-OS score compared with 47.8% in the non-ablation group (*Figure 1*; *P* <0.001**)**. In the multivariable logistic regression model using a complete case data set [440 (99.3%) of 443 patients], CA was significantly associated with a clinically meaningful improvement in the AFEQT-OS score [adjusted OR, 2.05 (95% CI: 1.14–3.68); *P* = 0.016 in *Table 2*]. A subgroup analysis showed that CA was significantly associated with a clinically meaningful improvements in the AFEQT-OS score in patients with LVEF ≥50% but not in those with LVEF <50% (*Table 3*), although the interaction with LVEF <50% or ≥50% was not significant (*P* for interaction = 0.746). When comparing the change in AFEQT-OS score as a continuous variable, their distributions were almost similar between patients with LVEF <50% and ≥50%: median (IQR), 10.5 (0.5–31.7) in the ablation group (*n* = 18) vs. 4.0 (−3.7 to 21.0) in the non-ablation group (*n* = 143), *P* = 0.188 for LVEF <50%; and 9.3 (4.6–19.5) in the ablation group (*n* = 41) vs. 4.4 (−5.1 to 14.8) in the non-ablation group (*n* = 207), *P* = 0.023 for LVEF ≥50%. Similar results were confirmed in the data sets from multiple imputation analyses using MAR.

**Table 2:** Association with clinical factors and improvements in health-related quality of life (complete case analyses)

Characteristic	Univariate			Multivariate		
	OR	95% CI	*P*-value	OR	95% CI	*P*-value
Catheter ablation						
ȃYes	2.28	1.30–3.99	0.004	2.05	1.14–3.68	0.017
ȃNo	1.00	Reference		1.00	Reference	
Sex						
ȃMale	0.94	0.64–1.38	0.751	1.15	0.71–1.86	0.561
ȃFemale	1.00	Reference		1.00	Reference	
Type of atrial fibrillation						
ȃParoxysmal	0.85	0.55–1.32	0.474	1.12	0.69–1.83	0.651
ȃOthers	1.00	Reference		1.00	Reference	
Coronary artery disease						
ȃYes	0.92	0.55–1.55	0.761	0.93	0.52–1.67	0.809
ȃNo	1.00	Reference		1.00	Reference	
Age						
ȃ1-year increment	0.99	0.97–1.00	0.137	0.99	0.97–1.01	0.174
AFEQT-OS score at baseline						
ȃ1-point increment	0.93	0.91–0.95	<0.001	0.93	0.91–0.94	<0.001

OR, odds ratio; CI, confidence interval; AFEQT-OS, Atrial Fibrillation Effect on QualiTy of life-Overall Summary.

**Table 3:** Subgroup analysis to assess associations with clinical factors and improvements in health-related quality of life stratified by LVEF

Multivariate analysis	LVEF <50%			LVEF ≥50%		
	OR	95% CI	*P*-value	OR	95% CI	*P*-value
Catheter ablation						
ȃYes	1.52	0.48–4.83	0.476	2.52	1.25–5.08	0.009
ȃNo	1.00	Reference		1.00	Reference	
Sex						
ȃMale	1.97	0.80–4.86	0.14	0.84	0.48–1.48	0.554
ȃFemale	1.00	Reference		1.00	Reference	
Type of atrial fibrillation						
ȃParoxysmal	1.66	0.58–4.79	0.346	0.87	0.49–1.55	0.642
ȃOthers	1.00	Reference		1.00	Reference	
Coronary artery disease						
ȃYes	0.57	0.19–1.76	0.332	1.28	0.63–2.58	0.492
ȃNo	1.00	Reference		1.00	Reference	
Age						
ȃ1-year increment	0.99	0.96–1.02	0.546	0.99	0.96–1.01	0.276
AFEQT-OS score at baseline						
ȃ1-point increment	0.90	0.87–0.93	<0.001	0.94	0.92–0.96	<0.001

OR, odds ratio; CI, confidence interval; AFEQT-OS, Atrial Fibrillation Effect on QualiTy of life-Overall Summary.

After the sensitivity analysis excluding 103 patients without a prescription for diuretics at baseline [340 (76.7%) of 443 patients), a positive association with CA and a clinically meaningful improvement in the AFEQT-OS score was consistently observed. However, this association was not statistically significant [adjusted OR, 2.18 (95% CI: 0.95–3.70); *P* = 0.069 in Supplementary material online, *Table S1*].

In the ablation group, 72 (96.0%) patients responded to the AFEQT questionnaire at both the baseline and 1-year visits. Of these, 69 patients had a single 12-lead ECG recorded at the 1-year follow-up. When successful CA was defined as sinus rhythm on ECG and being unaware of having an episode of AF within 1 month on the AFEQT questionnaire at follow-up, there was no significant difference in successful CA between patients with LVEF <50% and ≥50% (65.0% vs. 74.3%; *P* = 0.466).

### Cardiovascular events

**Figure 2:**
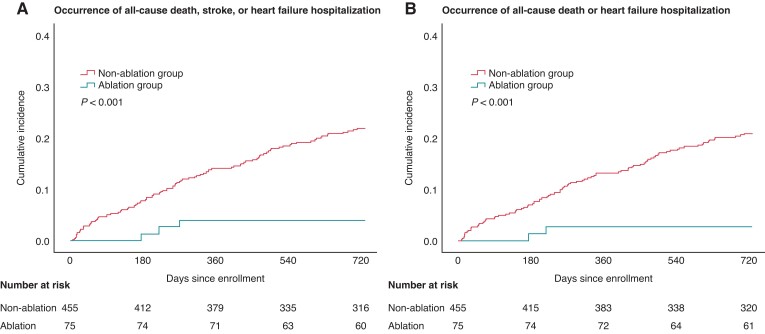
Unadjusted Kaplan–Meier curves for the composite of (*A*) all-cause death, stroke, or heart failure hospitalization, and (*B*) all-cause death or heart failure hospitalization.

During the mean (standard deviation) follow-up periods of 665 (161) days, the composite endpoint of all-cause death, stroke, or HF hospitalization occurred less frequently in 4.0% (*n* = 3) of patients in the ablation group vs. 20.9% (*n* = 95) in the non-ablation group (*P* < 0.001 for log-rank test; *Figure 2A*). All-cause death or HF hospitalization incidence was also less frequent in the ablation group than in the non-ablation group (*P* < 0.001; *Figure 2B*). In the univariable model, AF type (i.e. paroxysmal vs. persistent vs. others) was not associated with cardiovascular events. In the multivariable models using a complete case data set [473 (89.2%) of 530 patients], CA remained significantly associated with lower incidence in both composite endpoints (adjusted HRs for the composite of all-cause death, stroke, or HF hospitalization, 0.30 (95% CI: 0.09–0.96); *P* = 0.041; and for the composite of all-cause death or HF hospitalization, 0.21 (95% CI: 0.05–0.88); *P* = 0.033; *Table 4*). The multivariable models using multiple imputation data sets showed results similar to those of the complete case analyses (see Supplementary material online, *Table S2*). Despite adding B-type natriuretic peptide levels into the multivariable models, the results from the multiple imputation data sets demonstrated that CA was associated with a decrease in both composite endpoints [adjusted HRs for the composite of all-cause death, stroke, or HF hospitalization, 0.28 (95% CI: 0.09–0.91); *P* = 0.034; and for the composite of all-cause death or HF hospitalization, 0.21 (95% CI: 0.05–0.85); *P* = 0.028]. Furthermore, adding each variable such as age, sex, hypertension, diabetes mellitus, stroke, and vascular disease individually into the multivariable model (instead of the CHA_2_DS_2_-VASc score) yielded estimates that were consistent with the aforementioned analysis (see Supplementary material online, *Table S3*).

**Table 4:** Multivariable Cox proportional hazards models for each composite endpoint (complete case analyses)

Variables	All-cause death, stroke, or heart failure hospitalization			All-cause death or heart failure hospitalization		
	HR	95% CI	*P*-value	OR	95% CI	*P*-value
Catheter ablation						
ȃYes	0.30	0.09–0.95	0.041	0.21	0.05–0.88	0.033
ȃNo	1.00	Reference		1.00	Reference	
Type of atrial fibrillation						
ȃParoxysmal	1.03	0.63–1.68	0.913	1.12	0.68–1.83	0.66
ȃOthers	1.00	Reference		1.00	Reference	
Renal function						
ȃeGFR < 60 mL/min/1.73 m^2^	1.13	0.69–1.85	0.618	1.09	0.66–1.79	0.741
ȃeGFR ≥ 60 mL/min/1.73 m^2^	1.00	Reference		1.00	Reference	
Anaemia						
ȃPresent	2.02	1.31–3.13	0.002	1.98	1.27–3.09	0.003
ȃAbsent	1.00	Reference		1.00	Reference	
CHA_2_DS_2_-VASc score						
ȃ1-point increment	1.32	1.14–1.52	<0.001	1.38	1.19–1.60	<0.001
LVEF						
ȃ1% increment	0.98	0.96–0.99	0.005	0.97	0.95–0.99	<0.001

HR, hazard ratio; CI, confidence interval; eGFR, estimated glomerular filtration rate; LVEF, left ventricular ejection fraction.

In the sensitivity analysis excluding patients without a prescription for diuretics at baseline, CA was consistently associated with a lower event rate for both composite endpoints (see Supplementary material online, *Table S4*).

## Discussion

In the present study, the associations between CA and both health-related QoL and cardiovascular events were evaluated in consecutive patients with concomitant AF and HF, many of whom had preserved LVEF. At 1 year after study enrolment, more patients in the ablation group improved health-related QoL than those in the non-ablation group. We also observed a lower risk of cardiovascular events, including all-cause death, stroke, and HF hospitalization, in the ablation group than in the non-ablation group.

Our study found a significant association between the use of CA and improvements in health-related QoL in HF patients with mildly reduced or preserved LVEF, consistent with a sub-analysis of the CABANA trial.^8^ Compared with the CABANA trial, the patients in our study were older and had a lower body mass index, worse renal function, and a higher CHA_2_DS_2_-VASc score. However, the median period since the onset of AF was shorter (0.3 years vs. 1.1 years: see Supplementary material online, *Table S5*). This association was not observed in the patients with LVEF <50% in the subgroup analysis, although the point estimation tended toward a positive association with CA and improvements in health-related QoL. Several studies have previously reported that CA improves QoL in patients with concomitant AF and HFrEF.^18–21^ Furthermore, a retrospective single-centre cohort study reported that CA had similar effectiveness in functional status and symptoms among patients with both HFrEF and HFpEF.^10^ In a clinical setting involving many patients that were older and had a high baseline risk, our findings indicate that the favourable association between CA and patient outcomes is applicable to a broader range of early stage AF patients with HF who are referred to cardiology subspecialty clinics.

In the contemporary era, assessment of health-related QoL plays a key role in cardiovascular care. However, there is no consensus on which QoL assessment tools are most relevant in AF patients complicated with HF. In the aforementioned studies, Short Form 36 (SF-36) was used to assess health-related QoL.^18,19^ SF-36 is perhaps the most common generic QoL measure and helps determine the overall QoL in the population. However, generic QoL measures may not be as sensitive to the effect of a single disease on QoL.^22^ In contrast, disease-specific questionnaires help assess domains more relevant to a particular condition and may provide more targeted information to facilitate shared decision-making. The Kansas City Cardiomyopathy Questionnaire (KCCQ) is a validated questionnaire for assessing HF-specific QoL across a broad spectrum of HF patients, even in those with complicated AF.^23^ Further studies are warranted to evaluate which disease-specific questionnaires, AFEQT or KCCQ, should be used to assess health-related QoL among patients with concomitant AF and HF.

As for cardiovascular events, our findings were consistent with those of representative trials.^6,8^ Most patients in our cohort had LVEF >35%, and as such, this study verified the results from prior studies in patients with a higher LVEF range.^6,24^ From the standpoint of pathophysiological mechanisms, fibrosis, stretching, denervation, and natriuretic peptide depletion in the left atrium in AF can exacerbate HFrEF and HFpEF.^12^ However, other causal relationships between HF and AF are likely to differ between HFrEF and HFpEF and should be assessed separately. Neurohormonal activation is more significant in HFrEF and may be further exacerbated by the fast heart rate and irregularity of AF. In contrast, inflammation that may predispose a patient to AF may initially be more associated with the metabolic milieu of HFpEF, but immune activation increases with disease severity in both HFrEF and HFpEF. Therefore, mechanistic studies are needed to characterize AF phenotypes that are specific to HFpEF and HFrEF. Although different therapeutic approaches may be needed, CA is likely to improve hemodynamics regardless of baseline LVEF, which may be related to symptoms, health-related QoL, and cardiovascular events. However, a sub-analysis of the CABANA trial suggested that CA did not reduce HF hospitalization.^8^ Meanwhile, the EAST-AFNET4 trial showed early rhythm control with antiarrhythmic agents, and CA effectively reduced all-cause death or HF hospitalization.^9^ The duration of AF may be important to account for the difference in prognostic benefits of CA in patients with AF complicated by HF. The duration of AF in our cohort was as short as that in the EAST-AFNET4 trial (median, 0.3 years vs. 0.1 years). In contrast, the patients in the CABANA trial had a longer duration of AF (median, 1.1 years) (see Supplementary material online, *Table S5*).^8,9^ A meta-analysis including six observational studies reported that the time from initial AF diagnosis to CA was significantly associated with lower ablation success rates and higher AF recurrence rates: a diagnosis-to-ablation time of 1 year or less was associated with a 27% lower risk in AF recurrence post-ablation.^25^ In fact, ablation success rates for patients complicated with HF in our cohort was considered higher than that of the previous study composed of patients with and without HF.^25^ Replication in a large-scale database and/or randomized controlled trial is required to refine the clinical significance of CA in patients with mildly reduced and preserved LVEF.

### Limitations

Our results should be considered in light of several limitations. First, our registry consists of only 11 institutions within the Kanto region of Japan, and the results may not be generalizable to other countries or regions in Japan. However, patient characteristics and demographics in our cohort were similar to those in other international registries.^26^ Second, HF was defined phenotypically (i.e. signs and symptoms of HF, echocardiography, and natriuretic peptide level) by individual cardiologists without the need for confirmatory testing. Third, the limited number of patients and clinical events resulted in relatively low power to detect the studied outcomes, especially cardiovascular events. In addition, we did not perform substantial statistical adjustments using multivariable models or other methods, such as propensity score matching. Fourth, in the outpatient setting after enrolment, HF medication (i.e. diuretics and disease-modifying drugs) as well as exercise therapy could be adjusted according to individual signs and symptoms of HF; however, such data were not available in this study. Finally, unknown confounding factors may have influenced our results.

## Conclusion

In the absence of sufficient evidence for optimal rhythm control strategies with antiarrhythmic drugs and CA, current practice guidelines for the management of HF recommend CA of AF to relieve symptoms of HF in patients with persistent symptoms of HF despite medical therapy. Under these circumstances, our analysis using a large-scale cohort study showed that CA relative to drug therapy was significantly associated with improved health-related QoL and reduced incidence of cardiovascular events among patients with AF and a mildly reduced and preserved LVEF. To confirm the symptomatic and prognostic benefits of CA therapy, large-scale randomized controlled trials are needed across the clinical profile spectrum in patients with concomitant AF and HF, especially those with mildly reduced and preserved LVEF.

## Supplementary material

Supplementary material is available at *Europace* online.

## Supplementary Material

euac108_Supplementary_Data

## Data Availability

The data underlying this article will be shared on reasonable request to the corresponding author.
