# Supplementary material for: Catheter ablation for patients with atrial fibrillation and heart failure with reduced and preserved ejection fraction: insights from the KiCS-AF multicentre cohort study
Source: Europace. 2022 Jul 19;25(1):83–91. doi: 10.1093/europace/euac108 (PMC10103568; doi:10.1093/europace/euac108)
Supplement: euac108_Supplementary_Data [file euac108_supplementary_data.docx]

**Catheter Ablation for Patients with Atrial Fibrillation and Heart Failure with Reduced and Preserved Ejection Fraction: Insights from the KiCS-AF Multicenter Cohort Study**

**Supplementary Appendix**

Yasuyuki Shiraishi, MD, PhD,^1^ Shun Kohsaka, MD, PhD,^1^ Nobuhiro Ikemura, MD, PhD,^1^ Niimi Nozomi, MD,^1^ Takehiro Kimura, MD, PhD,^1^ Yoshinori Katsumata, MD, PhD,^1^

Kojiro Tanimoto, MD, PhD,^2^ Masahiro Suzuki, MD, PhD,^3^ Ikuko Ueda, PhD,^1^

Seiji Takatsuki, MD, PhD^1^ and Keiichi Fukuda, MD, PhD^1^

1. Department of Cardiology, Keio University School of Medicine, Tokyo, Japan
2. Department of Cardiology, National Hospital Organization Tokyo Medical Center, Tokyo, Japan
3. Department of Cardiology, National Hospital Organization Saitama National Hospital, Saitama, Japan

**Table of Contents**

**Table S1**. Sensitivity analysis on association with clinical factors and improvements in health-related quality of life in patients receiving diuretics.

**Table S2.** Multivariable Cox proportional hazards models for each composite endpoint (integrated results from multiple-imputation datasets [n = 10]).

**Table S3.** Multivariable Cox proportional hazards model including each variable comprising CHA_2_DS_2_-VASc score individually for the composite endpoint of all-cause death, stroke, or heart failure hospitalization (integrated results from multiple-imputation datasets [n = 10]).

**Table S4**. Sensitivity analysis for each composite endpoint in patients receiving diuretics (integrated results from multiple-imputation datasets [n = 10]).

**Table S5**. Baseline characteristics of the patients in our study and the CABANA sub-study.

Table S1. Sensitivity analysis on association with clinical factors and improvements in health-related quality of life in patients receiving diuretics

| Characteristic | Univariate | | | Multivariate | | |
| --- | --- | --- | --- | --- | --- | --- |
|  | OR | 95% CI | p value | OR | 95% CI | p value |
| Catheter ablation |  |  |  |  |  |  |
| Yes | 1.97 | 0.99–3.92 | 0.053 | 2.18 | 0.95–3.70 | 0.069 |
| No | 1.00 | Reference |  | 1.00 | Reference |  |
| Sex |  |  |  |  |  |  |
| Male | 0.87 | 0.79–1.55 | 0.523 | 1.09 | 0.65–1.85 | 0.740 |
| Female | 1.00 | Reference |  | 1.00 | Reference |  |
| Type of atrial fibrillation |  |  |  |  |  |  |
| Paroxysmal | 0.85 | 0.51–1.43 | 0.542 | 1.40 | 0.80–2.44 | 0.24 |
| Others | 1.00 | Reference |  | 1.00 | Reference |  |
| Coronary artery disease |  |  |  |  |  |  |
| Yes | 0.86 | 0.48–1.53 | 0.609 | 0.77 | 0.41–1.48 | 0.437 |
| No | 1.00 | Reference |  | 1.00 | Reference |  |
| Age |  |  |  |  |  |  |
| 1-year increment | 0.99 | 0.97–1.01 | 0.311 | 0.99 | 0.97–1.01 | 0.435 |
| AFEQT-OS score at baseline |  |  |  |  |  |  |
| 1-point increment | 0.94 | 0.92–0.95 | <0.001 | 0.93 | 0.92–0.95 | <0.001 |

Abbreviation: OR, odds ratio; CI, confidence interval; AFEQT-OS, Atrial Fibrillation Effect on Quality of Life-Overall Summary.

Table S2. Multivariable Cox proportional hazards models for each composite endpoint (integrated results from multiple-imputation datasets [n = 10])

| Variables | All-cause death, stroke, or heart failure hospitalization | | | All-cause death or heart failure hospitalization | | |
| --- | --- | --- | --- | --- | --- | --- |
|  | HR | 95% CI | p value | OR | 95% CI | p value |
| Catheter ablation |  |  |  |  |  |  |
| Yes | 0.28 | 0.09–0.89 | 0.031 | 0.20 | 0.05–0.83 | 0.026 |
| No | 1.00 | Reference |  | 1.00 | Reference |  |
| Type of atrial fibrillation |  |  |  |  |  |  |
| Paroxysmal | 1.04 | 0.65–1.65 | 0.887 | 1.10 | 0.68–1.77 | 0.709 |
| Others | 1.00 | Reference |  | 1.00 | Reference |  |
| Renal function |  |  |  |  |  |  |
| eGFR < 60 ml/min/1.73m^2^ | 1.13 | 0.70–1.83 | 0.62 | 1.06 | 0.65–1.74 | 0.814 |
| eGFR ≥ 60 ml/min/1.73m^2^ | 1.00 | Reference |  | 1.00 | Reference |  |
| Anemia |  |  |  |  |  |  |
| Present | 1.88 | 1.23–2.88 | 0.004 | 1.82 | 1.18–2.81 | 0.007 |
| Absent | 1.00 | Reference |  | 1.00 | Reference |  |
| CHA_2_DS_2_-VASc score |  |  |  |  |  |  |
| 1-point increment | 1.34 | 1.17–1.54 | <0.001 | 1.38 | 1.20–1.59 | <0.001 |
| LVEF |  |  |  |  |  |  |
| 1% increment | 0.98 | 0.96–1.00 | 0.015 | 0.97 | 0.96–0.99 | 0.003 |

Abbreviation: HR, hazard ratio; CI, confidence interval; eGFR, estimated glomerular filtration rate; LVEF, left ventricular ejection fraction.

Table S3. Multivariable Cox proportional hazards model including each variable comprising CHA_2_DS_2_-VASc score individually for the composite endpoint of all-cause death, stroke, or heart failure hospitalization (integrated results from multiple-imputation datasets [n = 10]).

| Variables | All-cause death, stroke, or heart failure hospitalization | | |
| --- | --- | --- | --- |
|  | HR | 95% CI | p value |
| Catheter ablation |  |  |  |
| Yes | 0.31 | 0.10–0.99 | 0.048 |
| No | 1.00 | Reference |  |
| Age |  |  |  |
| ≥ 75 years | 4.52 | 1.94–10.5 | <0.001 |
| 65–74 years | 1.83 | 0.79–4.23 | 0.158 |
| < 65 years | 1.00 | Reference |  |
| Sex |  |  |  |
| Male | 1.64 | 1.08–2.51 | 0.021 |
| Female | 1.00 | Reference |  |
| Hypertension |  |  |  |
| Yes | 1.00 | 0.64–1.56 | 0.988 |
| No | 1.00 | Reference |  |
| Diabetes mellitus |  |  |  |
| Yes | 1.07 | 0.67–1.71 | 0.78 |
| No | 1.00 | Reference |  |
| Stroke |  |  |  |
| Yes | 1.09 | 0.61–1.96 | 0.771 |
| No | 1.00 | Reference |  |
| Vascular disease |  |  |  |
| Yes | 1.65 | 1.03–2.63 | 0.037 |
| No | 1.00 | Reference |  |
| LVEF |  |  |  |
| <50% | 1.46 | 0.95–2.23 | 0.083 |
| ≥50% | 1.00 | Reference |  |
| Anemia |  |  |  |
| Yes | 1.68 | 1.09–2.59 | 0.019 |
| No | 1.00 | Reference |  |

Abbreviation: HR, hazard ratio; CI, confidence interval; LVEF, left ventricular ejection fraction.

Table S4. Sensitivity analysis for each composite endpoint in patients receiving diuretics (integrated results from multiple-imputation datasets)

| Variables | All-cause death, stroke, or heart failure hospitalization | | | All-cause death or heart failure hospitalization | | |
| --- | --- | --- | --- | --- | --- | --- |
|  | HR | 95% CI | p value | OR | 95% CI | p value |
| Catheter ablation |  |  |  |  |  |  |
| Yes | 0.25 | 0.06–1.02 | 0.053 | 0.14 | 0.02–0.98 | 0.048 |
| No | 1.00 | Reference |  | 1.00 | Reference |  |
| Type of atrial fibrillation |  |  |  |  |  |  |
| Paroxysmal | 1.28 | 0.78–2.10 | 0.322 | 1.36 | 0.82–2.27 | 0.234 |
| Others | 1.00 | Reference |  | 1.00 | Reference |  |
| Renal function |  |  |  |  |  |  |
| eGFR < 60 ml/min/1.73m^2^ | 1.00 | 0.60–1.68 | 0.99 | 0.95 | 0.56–1.61 | 0.848 |
| eGFR ≥ 60 ml/min/1.73m^2^ | 1.00 | Reference |  | 1.00 | Reference |  |
| Anemia |  |  |  |  |  |  |
| Present | 1.92 | 1.23–3.01 | 0.004 | 1.85 | 1.17–2.92 | 0.008 |
| Absent | 1.00 | Reference |  | 1.00 | Reference |  |
| CHA_2_DS_2_-VASc score |  |  |  |  |  |  |
| 1-point increment | 1.37 | 1.18–1.58 | <0.001 | 1.41 | 1.22–1.64 | <0.001 |
| LVEF |  |  |  |  |  |  |
| 1% increment | 0.98 | 0.97–1.00 | 0.087 | 0.98 | 0.96–1.00 | 0.023 |

Abbreviation: HR, hazard ratio; CI, confidence interval; eGFR, estimated glomerular filtration rate; LVEF, left ventricular ejection fraction.

Table S5. Baseline characteristics of the patients in our study and the CABANA sub-study

|  | Our study  n = 530 | CABANA sub-study  n = 778 |
| --- | --- | --- |
| Age, years | 74 (66–80) | 68 (62–73) |
| Men, n (%) | 325/530 (61.3%) | 433/778 (55.7%) |
| Body mass index, kg/m^2^ | 23 (20–25) | 31 (27–35) |
| LVEF, % | 55 (41-60) | 55 (50–61) |
| LVEF ≤ 35%, n (%) | 81/490 (16.5%) | 45/571 (7.9%) |
| AF duration, years | 0.3 (0.1–1.9) | 1.1 (0.3–4.1) |
| Type of AF |  |  |
| Paroxysmal, n (%) | 125/526 (23.8%) | 246/778 (31.6%) |
| Persistent, n (%) | 193/526 (36.7%) | 430/778 (55.3%) |
| Longstanding persistent / Permanent, n (%) | 181/526 (34.4%) | 102/778 (13.1%) |
| Comorbidities |  |  |
| Coronary artery disease, n (%) | 78/530 (14.7%) | 170/778 (21.9%) |
| Hypertension, n (%) | 355/530 (67.0%) | 665/778 (85.5%) |
| Diabetes mellitus, n (%) | 127/530 (24.0%) | 195/778 (25.1%) |
| Stroke, n (%) | 60/530 (11.3%) | 79/778 (10.2%) |
| CKD (GFR < 60 ml/min/1.73m^2^), n (%) | 343/519 (66.1%) | 181/778 (19.8%) |
| CHA_2_DS_2_-VASc score | 4 (3–5) | 3 (2–4) |

Abbreviation: HF, heart failure; LVEF, left ventricular ejection fraction; AF, atrial fibrillation; CKD, chronic kidney disease; GFR, glomerular filtration rate.
